# Diversity and Pathogenicity of *Colletotrichum* Species Causing Coffee Anthracnose in China

**DOI:** 10.3390/microorganisms13030512

**Published:** 2025-02-26

**Authors:** Ying Lu, Weiyi Zhang, Xiaoli Hu, Chunping He, Yanqiong Liang, Xing Huang, Kexian Yi, Weihuai Wu

**Affiliations:** 1 Environment and Plant Protection Institute, Chinese Academy of Tropical Agricultural Sciences, Key Laboratory of Integrated Pest Management on Tropical Crop, Ministry of Agriculture and Rural Affairs, Haikou 571101, China; ytluy2010@163.com (Y.L.); huangxing@catas.cn (X.H.);; 2 Hainan Key Laboratory for Monitoring and Control of Tropical Agricultural Pests, Haikou 571101, China; 3 School of Tropical Agricultural and Forestry, Hainan University, Danzhou 571737, China; 4 College of Plant Science and Technology, Huazhong Agricultural University, Wuhan 430070, China; 5 Sanya Research Institute, Chinese Academy of Tropical Agricultural Sciences, Sanya 572025, China

**Keywords:** coffee anthracnose, *Colletotrichum* spp. pathogenicity tests, diagnosis, taxonomy

## Abstract

Coffee is a significant traded commodity for developing countries. Among the various diseases affecting coffee, anthracnose caused by *Colletotrichum* spp. has re-emerged as a major constraint on global coffee production. To better understand the *Colletotrichum* species complex associated with coffee anthracnose, we characterized *Colletotrichum* spp. using a combination of phenotypic traits, *MAT1-2* (ApMat) gene analysis, multi-locus phylogenetic (*ITS*, *ACT*, *CHS-1*, and *GAPDH*), and pathogenicity assays. A total of 74 *Colletotrichum* isolates were collected from coffee plants exhibiting anthracnose symptoms across nine coffee plantations in China. Among these, 55 isolates were identified as the *C. gloeosporioides* species complex using the *ApMat* locus, while the remaining 19 isolates were identified through multi-locus phylogenetic analyses. The isolates represented seven *Colletotrichum* species from five species complexes: *C. gloeosporioides* (including *C. siamense*, *C. nupharicola*, and *C. theobromicola*), *C. boninens* (*C. karstii*), *C. gigasporum* (*C. gigasporum*), *C. orchidearum* (*C. cliviicola*), and *C. magnum* (*C. brevisporum*). This is the first report of *C. nupharicola* and *C. cliviicola* causing coffee anthracnose worldwide, and the first report of *C. nupharicola* in China. Pathogenicity tests confirmed that all seven species were capable of infecting coffee leaves. This research enhances our understanding of the *Colletotrichum* species responsible for coffee anthracnose, and provides valuable insights for developing effective disease management strategies.

## 1. Introduction

Coffee is a major traded commodity in the developing world [1]. Three primary commercial varieties are cultivated globally: *Coffea arabica* (*arabica*), *C. canephora* var. *robusta* (*robusta*), and *C. liberica* (*liberica*) [2]. Among these, arabica and robusta are the dominant species, accounting for over 99% of global exports. Coffee was introduced to China more than a century ago, and has since become a significant cash crop in Yunnan and Hainan provinces. *Arabica* coffee is predominantly cultivated in Yunnan, while *robusta* coffee is mainly grown in Hainan [1,3].

Diseases are among the most critical factors limiting coffee production. The most prevalent and economically significant diseases include coffee rust (*Hemileia vastatrix*), Cercospora leaf spot (*Cercospora coffeicola*), Phoma leaf spot (*Phoma* spp.), and anthracnose and blister spot caused by *Colletotrichum* spp. Coffee anthracnose, caused by species of the genus *Colletotrichum*, is a major issue in Southeast Asia, India, and China [4,5,6]. Anthracnose is characterized by irregular, large brown-to-gray patches that often appear along the edges of leaves. Other symptoms may include dieback, brown blight, and blister spots, which manifest as light-green, oily-looking lesions on both leaves and fruit [6,7]. The genus *Colletotrichum* is a significant group within the fungal family Glomerellaceae, order Glomerellales, class Sordariomycetes, and phylum Ascomycota. It is recognized as one of the top 10 most important genera of plant pathogenic fungi globally, with a wide range of impacts on agriculture and ecosystems [8]. *Colletotrichum* species are notorious for causing anthracnose diseases in a wide variety of plants, leading to significant agricultural losses [9,10,11,12,13,14]. The genus *Colletotrichum* is highly diverse and complex, with its classification system continually evolving as new species are discovered and molecular techniques improve. A species complex (or ‘aggregate’) in *Colletotrichum* is defined as a monophyletic group of species that share common characteristics, such as similar conidial morphology. The current classification system recognizes 15 species complexes: *C. acutatum*, *C. agaves*, *C. boninense*, *C. caudatum*, *C. dematium*, *C. destructivum*, *C. dracaenophilum*, *C. gigasporum*, *C. gloeosporioides*, *C. graminicola*, *C. magnum*, *C. orbiculare*, *C. orchidearum*, *C. spaethianum*, and *C. truncatum*. Additionally, there are several singleton species that do not belong to any of these complexes. The *C. caudatum* species complex was initially considered a sub-aggregate within the *C. graminicola* complex, but is now often treated as a separate complex. Recently, Bhunjun et al. suggested merging the two into a single *C. graminicola*–*caudatum* species complex [15]. The phylogenetic backbone of *Colletotrichum* has been continuously updated as new species have been described: Cannon et al. [16]: 119 species; Jayawardena et al. [17] and Marin-Felix et al. [18]: 189 species; Jayawardena et al. [19]: 247 species; Bhunjun et al. [15]: 248 species. The discovery of new species highlights the high species diversity within the genus. Twenty *Colletotrichum* species from six species complexes have been reported to be associated with coffee worldwide, including *C. acutatum*, *C. costarricense*, *C. cuscutae*, and *C. walleri* from the *C. acutatum* species complex; *C. endophytica*, *C. ledongense*, *C. tropicale*, *C. asianum*, *C. fructicola*, *C. gloeosporioides*, *C. kahamae* subsp. *kahawae*, *C. queeslandicum*, *C. theobromicola* (syn. *C. fragariae*), and *C. siamense* from the *C. gloeosporioides* species complex; *C. boninense* and *C. karsitii* from the *C. boninense* species complex; *C. truncatum* (syn. *C. capsici*) from the *C. truncatum* species complex; *C. gigasporum* and *C. vietnamense* from the *C. gigasporum* species complex; and one singleton species (*C. brevisporum*) [13,20,21,22,23,24,25,26].

The exact *Colletotrichum* species causing diseases in coffee varies among regions. For example, *C. asianum*, *C. fructicola*, and *C. siamense* have been reported in northern Thailand [22]; *C. acutatum*, *C. boninense*, *C. truncatum*, *C. gigasporum*, *C. gloeosporioides*, *C. karstii*, *C. vietnamense*, and *C. walleri* in Vietnam [13,21]; *C. gigasporum*, *C. gloeosporioides*, *C. karstii*, *C. siamense*, and *C. theobromicola* in Mexico [27]; and *C. endophytica*, *C. fructicola*, *C. ledongense*, *C. siamense*, *C. tropicale*, *C. karstii*, *C. gigasporum*, and *C. brevisporum* in Hainan, China [26].

Sutton et al. suggested that relationships within the genus *Colletotrichum* were unlikely to be resolved using morphology alone [28]. Morphological plasticity and overlapping phenotypes make traditional taxonomic criteria unreliable for the accurate delineation of *Colletotrichum* species [29,30]. The adoption and use of multi-locus phylogenetic analysis, a polyphasic approach combining the application of molecular methods with morphological and pathogenicity data, significantly changed the classification and species concepts in *Colletotrichum* [20,25,31,32,33,34]. Therefore, this study aimed to identify *Colletotrichum* species on diseased coffee leaves in Hainan and Yunnan based on multi-gene phylogenetic analyses and morphological characteristics. The pathogenicity of different *Colletotrichum* species on coffee leaves was then assessed.

## 2. Materials and Methods

### 2.1. Sample Collection, Isolation, and Purification

In 2019, coffee leaves with typical anthracnose symptoms were collected from nine coffee plantations in Hainan and Yunnan, China. Seven Yunnan coffee plantations were arabica, and two plantations of Hainan were mixed plantations with both arabica and robusta. A total of 120 diseased coffee samples were obtained for fungal isolation (Table 1). The fungus was isolated from diseased samples utilizing the method delineated by Huang et al. [35]. Diseased tissues were surface-sterilized in 70% ethanol for 30 s, then in 1% NaClO for 1 min, before being rinsed in sterile distilled water for 30 s and dried on sterile paper (one piece of paper for each sample). Three 5 × 5 mm pieces of tissue taken from the margin of diseased tissues from each sample were plated onto potato dextrose agar (PDA; 200 g potato, 20 g glucose, 20 g agar, distilled water to 1 L), and incubated at 25 °C, with a 12 h photoperiod, for 2 weeks. After 20 days, colonies with asexual conidia developed on the edge of the leaf disk. Single-spore isolates were produced by diluting the conidia and spreading them on water agar, and later transferring single germination conidia to PDA. A total of 74 pure cultures were secured and were stored in sterilized water in microtubes at 4 °C, and stock cultures were stored in PDA slants at 4 °C in the dark; they were then deposited at the Environment and Plant Protection Institute, Chinese Academy of Tropical Agricultural Sciences.

### 2.2. DNA Extraction, PCR Amplification, and Sequencing

All isolates were grown on PDA for 7 days, at 25 °C, with a 12 h photoperiod. A small amount of aerial mycelium was scraped with a sterile 10 µL pipette tip from the colony surface. Genomic DNA of all isolates was extracted using the E.Z.N.A. Fungal DNA Mini Kit (Omega Bio-tek, Dusseldorf, Germany), following the manufacturer’s instructions.

ITS, actin (*ACT*), chitin synthase 1 (*CHS*-1), and the glycolytic enzyme glyceraldehyde 3-phosphate dehydrogenase (*GAPDH*) and the mating type locus MAT1-2 (*ApMat*) regions were amplified using the primer pairs in Table 2. PCR was performed using the Premix TaqTM (TaKaRa Taq^TM^ Version 2.0 plus dye) on a Heal Force thermal cycler T960 (Thermo Fisher Scientific, Waltham, MA, USA) in a 25 μL reaction volume. The PCR mixtures contained 1 μL of DNA template, 12.5 μL of the Premix Taq^TM^ (TaKaRa Taq^TM^ Version 2.0 plus dye), and 0.5 μL of 20 μM of each primer. PCR reactions for ITS were performed using the following conditions: initial denaturation at 95 °C for 5 min, followed by 35 cycles, each consisting of 45 s at 95 °C, 30 s at 55 °C, and then extension for 1 min at 72 °C, with a final extension step at 72 °C for 10 min. PCR conditions for other loci were the same, except for the annealing temperatures: *ACT*, *CHS-1*, and *GAPDH* at 58 °C, *GS* at 60 °C, and *ApMat* at 62 °C. PCR products were examined by electrophoresis in 1.2% agarose gels stained with GoodView Nucleic Acid Stain (Beijin SBS Genetech, Beijing, China) in 1 × Tris-acetate acid EDTA (TAE) buffer, and photographed under UV light. The amplicons were purified using the E.Z.N.A MicroElute Cycle Pure Kit and then sequenced by Invitrogen Company, Waltham, MA, USA.

### 2.3. Phylogenetic Analysis

Fungal sequences with high similarities to the gene/region sequences (*ITS*, *ACT*, *CHS-1*, *GAPDH*, and *ApMat*) of *Colletotrichum* species were identified and retrieved from the NCBI database (https://www.ncbi.nlm.nih.gov/). For each gene/region, sequences from pathogens belonging to the same species complex were aligned by the MAFFT v.7 online server [39]. The aligned sequences were manually edited using MEGA v.10 to improve the alignment [40]. All gaps were treated as missing data. Phylogenetic analyses were conducted based on concatenated loci for *Colletotrichum* species in MEGA v.10, using the maximum-likelihood method with the Tanmura-Nei model and 1000 bootstrap replicates, and figures of trees were created. The sequences derived in this study were deposited in GenBank (https://www.ncbi.nlm.nih.gov/) and NGDC (https://ngdc.cncb.ac.cn/) (Supplementary File: Tables S1 and S2).

### 2.4. Morphological Analysis

A morphological study of one isolate randomly selected from each *Colletotrichum* species (BSC4-1, BSC1-2, BSC1-3, Bai1, BEC191A, BEC92, and FS4-2) was carried out, following the procedures of Wire et al. [25]. Mycelial blocks (6 mm in diameter) were placed in the center of the plates. Each mycelial block was transferred onto four PDA plates that were subsequently incubated at 25 °C in the dark. Colony diameters were measured daily for 5 days to calculate the mycelial growth rates (mm/day). Characteristics of fungal colonies were recorded and colony colors were identified. Fungal structures that developed on the plant tissues or agar plates, Appressoria were produced using the glass culture technique, using a glass slide. The sizes and shapes of conidia, appressoria, and setae of acervuli were measured in >30 samples using an Olympus BX51 compound light microscope (Olympus America, Center Valley, PA, USA) fitted with a QImaging Retiga 2000R camera (Q Imaging, Surrey, BC, Canada).

### 2.5. Pathogenicity Assay

Twelve *Colletotrichum* isolates were identified based on phylogenetic analyses of multiple loci, and selected for pathogenicity testing by inoculating conidial suspension onto detached leaves. These comprised two *C. brevisporum* (BSC92, BSC15-2), *C. cliviicola* (FS4-2), one isolate from *C. gigasporum* (BEC191A), *C. karstii* (Bai 1, BEC26C), two *C. nupharicola* (BSC1-2, BEC106B), two *C. siamense* (BSC4-1, HG 9), and *C. theobromicola* (BSC1-3, IG3). For in vitro inoculation, asymptomatic young leaves of coffee were collected from 1-year-old coffee plants. Detached leaves were surface-sterilized with 75% ethanol, washed three times with sterile water, and air-dried on sterile filter paper. The leaves were wounded with a sterile needle (insect pin, 0.5 mm diameter); three wounds one either side of the midrib of each leaf were made. A 6 µL drop of conidial suspension (10^6^ conidia mL^−1^) was placed onto each wound on the left side of the leaf; similarly, sterile water was placed onto the wounds on the right side of the same leaf as a control. Treated leaves were then put in plastic trays, covered with a piece of plastic wrap to maintain a relative humidity, and incubated at 25 °C with a 12 h photoperiod, and monitored daily for lesion development. Symptoms in the coffee leaves were observed for 7 days after inoculation. The experiment followed a completely randomized design with three replicates per isolate, each replicate consisting of three leaves. The incidence of infection was calculated as (I_P_/N_P_) × 100, where I_P_ was the number of infected points on the leaves, and N_P_ was the number of inoculated points on the leaves. Pathogens were reisolated from the resulting lesions and identified as described above.

## 3. Results

### 3.1. Phylogenetic Analyses

We obtained 74 isolates of *Colletotrichum* spp. from diseased leaves of coffee plants from the main coffee growing region of China, and identified them based on phylogeny and morphological characteristics. Based on the BLAST (https://blast.ncbi.nlm.nih.gov/Blast.cgi, accessed on 15 December 2024) search results on the NCBI database with the ITS sequences, all *Colletotrichum* isolates in this study were preliminarily allocated to the following species complexes: the *C*. *gloeosporioides*, *C. gigasporum*, *C. orchidearum*, *C. magnum*, and *C. boninense* species complexes.

**Figure 1:**
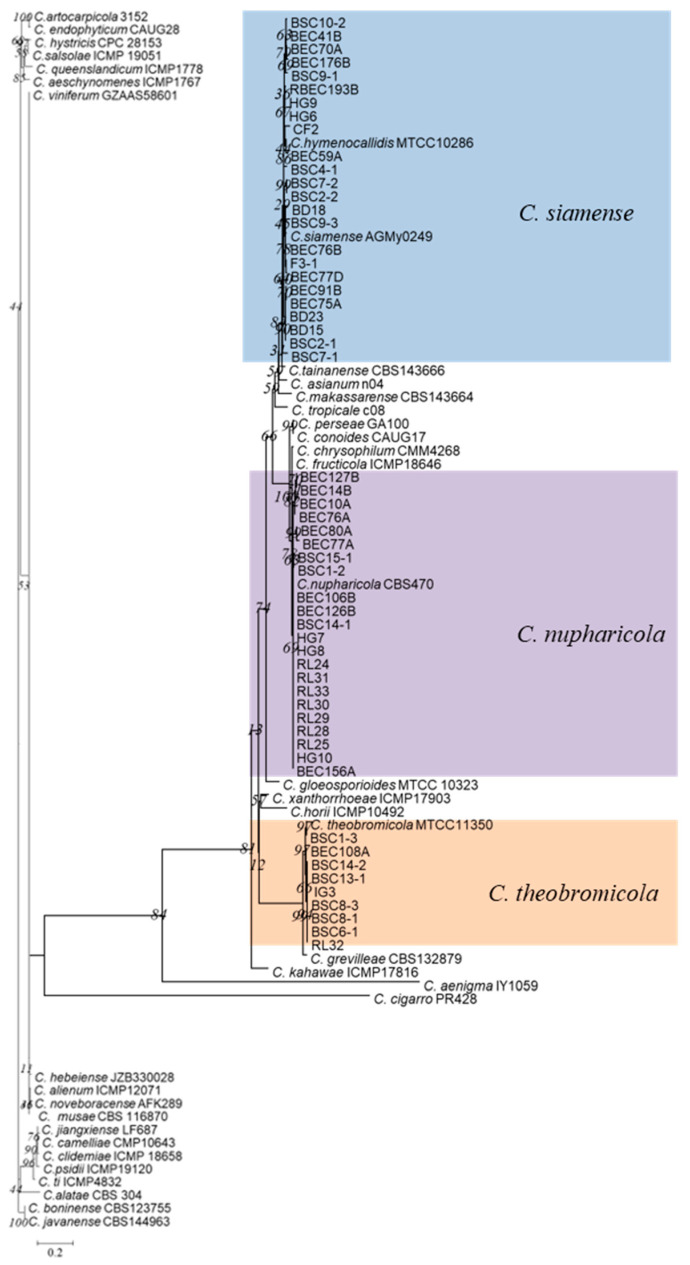
Phylogenetic tree generated by maximum likelihood analysis based on *ApMat* gene sequences from *Colletotrichum gloeosporioides* species complexes. Phylogeny is rooted with *C. boninense* (CBS 123755) and *C. javanense* (CBS144963) as outgroups.

The *ApMat* gene alignment contained 56 taxa, including *boninense* (CBS 123755) and *C. javanense* (CBS144963) as outgroups. The ML/BI phylogenetic analyses showed that 24 isolates were clustered into the same clade as *C*. *siamense*, 22 isolates were clustered into a clade with *C. nupharicola*, and 9 isolates were clustered with *C*. *theobromicola* (Figure 1).

**Figure 2:**
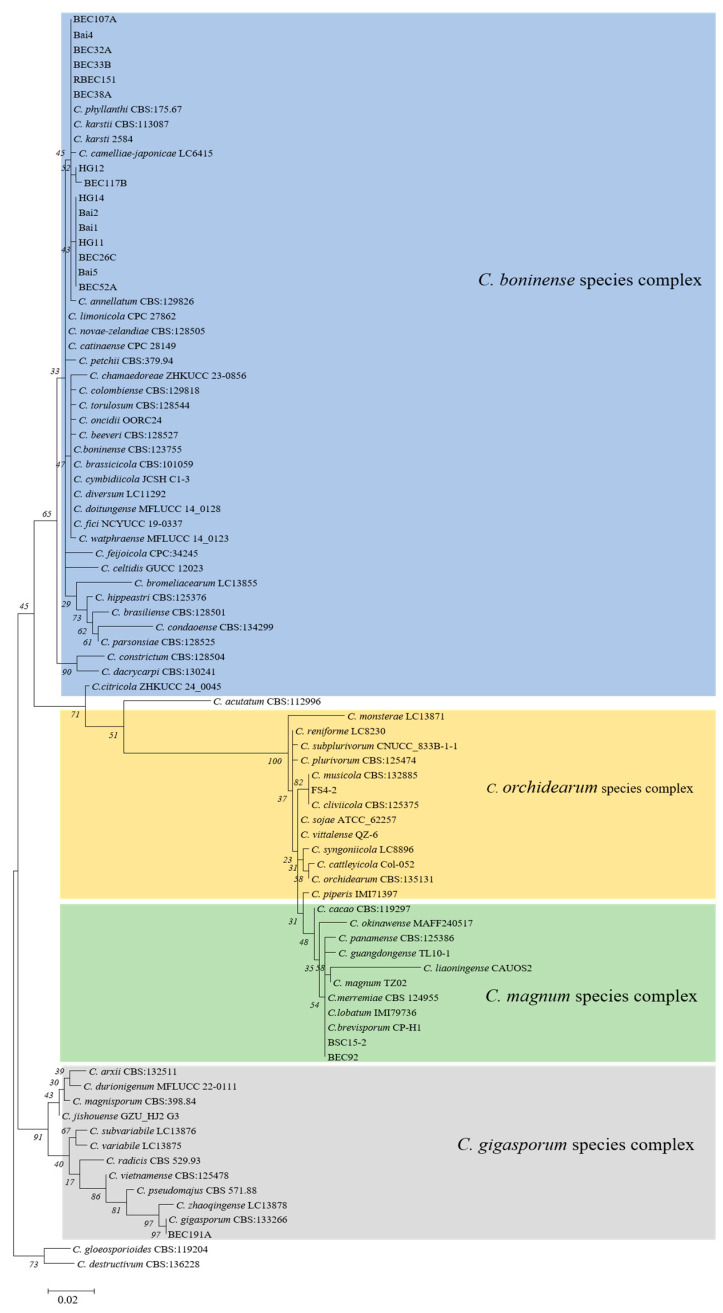
Phylogenetic tree generated by maximum likelihood analysis based on combined *ITS*, *ACT*, *CHS-1*, and *GAPDH* gene sequences. Tree displays phylogenetic relationships between *Colletotrichum* species isolated from coffee plants in China. *C. gloesporioides* (CBS:119204) and *C. destructivum* (CBS:136228) were used as outgroups.

Figure 2 demonstrates the phylogenetic relationship isolates in the *C. boninense*, *C. orchidearum*, *C. magnum*, and *C. gigasporum* species complexes. The concatenated alignment (*ITS*, *ACT*, *CHS-1*, and *GAPDH*) contained 19 isolates, with *C. gloesporioides* (CBS:119204) and *C. destructivum* (CBS:136228) as outgroups. The dataset comprised 1067 characters, including the alignment gaps. The gene boundaries in the alignment were *ITS*: 1-503, *ACT*: 504-739, *CHS-1*: 740-847, and *GAPDH*: 848-1067. A maximum of 1000 equally most parsimonious trees were retained. The trees generated from the PAUP and RAXML analyses were similar to that from the Bayesian analysis (Figure 2). Fifteen tested isolates belonging to *C. karstii* were clustered in the *C. boninense* species complex, one isolate (FS4-2) belonging to *C. cliviicola* was clustered in the *C. orchidearum* species complex, two isolates (BSC15-2 and BEC92) belonging to *C. brevisporum* were grouped in the *C. magnum* species complex, and one isolate (BEC191A) belonging to *C. gigasporum* was clustered in the *C. gigasporum* species complex (Figure 2).

### 3.2. Taxonomy

*Colletotrichum siamense* Prihastuti, L. Cai and K.D. Hyde, 2009 [29].

**Figure 3:**
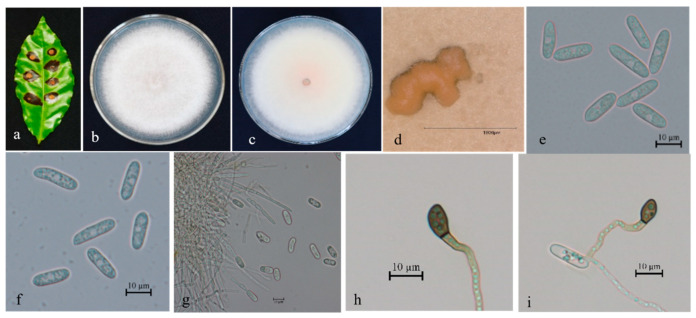
*Colletotrichum siamense* (BSC4-1) on leaf of host plant (**a**); surface (**b**) and reverse (**c**) sides of colony after incubation for 7 days on PDA; (**d**) conidiomata; (**e**,**f**) conidia; (**g**) conidiophore, conidiogenous cells, and conidia; (**h**,**i**) conidial appressoria. Scale bars: 10 µm (**e**–**i**).

Description. Colonies 63–72 mm in diameter after 7 days at 25 °C on PDA. Aerial mycelium is white and cottony, surface of colony has numerous orange conidiomata; reverse is white at first, then red at center. Conidia are hyaline, aseptate, smooth-walled, and ovoid to cylindrical, with both ends bluntly round, (11.9–) 14.1–19.7 (–23.3) × 3.8–4.9 μm, mean ± SD = 16.3 ± 1.4 × 4.1 ± 0.4 μm, L/W ratio = 3.9. Appressoria formed in slide culture: (8.5-) 10.1–12.9 (–14.5) × 4.1–6.0 μm, mean ± SD = 7.9 ± 1.7 × 5.1 ± 0.9 μm, L/W ratio = 1.8, formed from branched mycelia, terminal, brown-to-dark-brown, variable in shape, irregular. Sexual state not observed in culture (Figure 3).

Specimens examined. Diseased leaves on cultures BSC2-1, BSC2-2, BSC4-1, BSC7-1, BSC7-2, BSC9-1, BSC9-3, and BSC10-2 from Hainan, Baisha, China coffee planting base; on cultures BD15, BD18, and BD23 from Yunnan, Mangshi, Bandong coffee planting base; on cultures HG6 and HG9 from Yunnan, Mangshi, Hougu coffee base; on cultures BEC70A, BEC75A, BEC76B, and BEC77D from Nandao river, Yunnan province coffee planting base; on cultures BEC59A and BEC41B from Yunnan tropical crop institute demonstration base; on cultures BEC91B amd BEC176B from Yunnan agricultural germplasm resources nursery; on culture RBEC193B from Yunnan, Xinghua farm; and on cultures F3-1 and CF2 from Hainan, Fushan coffee planting base.

*Colletotrichum nupharicola* D.A. Johnson, Carris and J.D. Rogers, 1997 [41].

**Figure 4:**
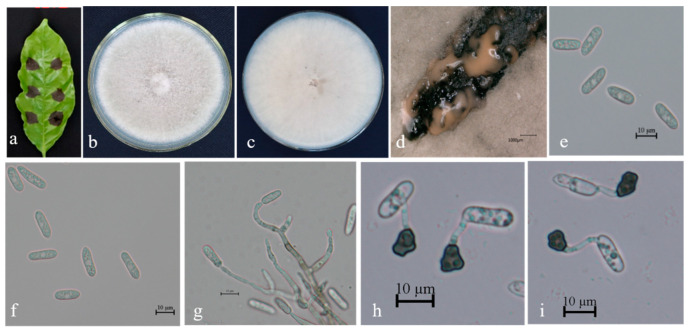
*Colletotrichum nupharicola* (BSC1-2) on leaf of host plant (**a**); surface (**b**) and reverse (**c**) sides of colony after incubation for 5 days on PDA; (**d**) conidiomata; (**e**,**f**) conidia; (**g**) conidiophore, conidiogenous cells, and conidia; (**h**,**i**) conidial appressoria. Scale bars: 10 μm (**e**–**i**).

Description. On PDA. Colonies 61–67 mm in diameter after 5 days at 25 °C. Aerial mycelium is dense, cottony, and white; reverse has a white, cottony surface. Conidiomata are apricot and black; vegetative hyphae are hyaline and medium-brown, usually smooth-walled, septate, and branched; chlamydospores and setae are not observed. Conidiophores are either directly formed from hyphae or from cushion of spherical, hyaline cells, septate and sometimes branched. Conidiogenous cells are hyaline-to-pale-brown, cylindrical, and straight to flexuous. Conidia are hyaline, usually aseptate, sometimes become 1–2 septate with age, are smooth-walled, are ovoid to cylindrical or clavate, with both ends rounded, or one end rounded and one end acute, are guttulate, and are granular; (12.5–) 14.0–19.7 (–21.3) × 3.8–4.9 μm, mean ± SD = 16.5 ± 1.2 × 4.3 ± 0.8 μm, L/W ratio = 3.7. Appressoria formed in slide culture: (5.3–) 6.2–8.5 (–9.3) × 4.0–6.1 μm, mean ± SD = 7.8 ± 0.8 × 5.2 ± 0.8 μm, L/W ratio = 1.2, formed from branched mycelia, terminal, brown-to-dark brown, variable in shape, irregular. Sexual state not observed in culture (Figure 4).

Specimens examined. Diseased leaves on cultures BSC1-2, BSC14-1, and BSC15-1 from China, Hainan, Baisha coffee planting base; on cultures HG7, HG8, and HG10 from Yunnan, Mangshi, Hougu coffee base; on cultures RL24, RL25, RL28, RL29, RL30, RL31, RL33, BEC80A, and BEC156A from Yunnan agricultural germplasm resources nursery; on cultures BEC76A and BEC77A from Nandao river, Yunnan province coffee planting base; on cultures BEC10A and BEC14B from Yunnan tropical crop institute demonstration base; and on cultures BEC106B, BEC126B, and BEC127B from Malipo, Wenshan, Yunnan coffee planting base.

*Colletotrichum theobromicola* Delacr. 1905.

Weir BS, Johnston PR, Damm U. 2012 [25].

**Figure 5:**
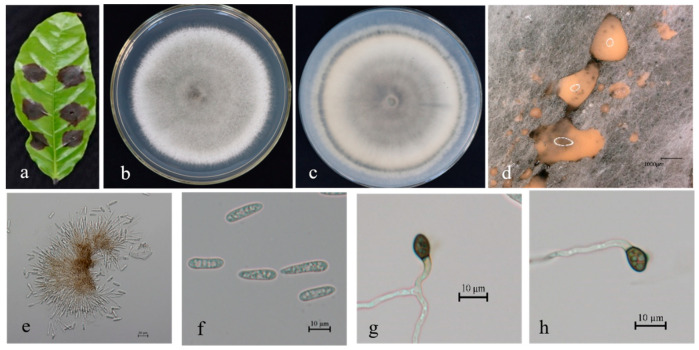
*Colletotrichum theobromicola* (BSC1-3) on leaf of host plant (**a**); surface (**b**) and reverse (**c**) sides of colony after incubation for 5 days on PDA; (**d**) conidiomata; (**e**) conidiophore, conidiogenous cells, and conidia; (**f**) conidia; (**g**,**h**) conidial appressoria. Scale bars: 10 μm (**e**–**h**).

Description. On PDA. Colonies raised, white-to-gray, dense aerial mycelium; reverse: gray-to-black, cottony surface of colony with numerous small orange/apricot conidiomata; colonies 62–69 mm in diameter after 5 days at 25 °C. Vegetative hyphae are hyaline-to-medium-brown, usually smooth-walled, septate, and branched; chlamydospores and setae not observed. Conidiophores are either directly formed from hyphae or from cushion of spherical, hyaline cells, septate, and sometimes branched. Conidiogenous cells are hyaline-to-pale-brown, cylindrical, and straight-to-flexuous. Conidia are hyaline, usually aseptate, smooth-walled, ovoid-to-cylindrical or clavate, with both ends rounded, or one end rounded and one end acute, and are guttulate and granular; (11.3–) 13.9–18.6 (–20.6) × 3.5–4.4 μm, mean ± SD = 16.3 ± 0.9 × 4.0 ± 0.5 μm, L/W ratio = 3.9. Appressoria formed in slide culture: (8.9–) 10.2–13.2 (–15.2) × 4.1-6.1 μm, mean ± SD = 7.6 ± 1.3 × 5.1 ± 0.4 μm, L/W ratio = 2.2, formed from branched mycelia, terminal, brown-to-dark-brown, variable in shape, and irregular. Sexual state not observed in culture (Figure 5).

Specimens examined. Diseased leaves on cultures BSC1-3, BSC6-1, BSC8-1, BSC8-3, BSC13-1, and BSC14-2 from China, Hainan, Baisha coffee planting base; on culture IG3 from Hainan, Fushan coffee planting base; on culture RL32 from Yunnan agricultural germplasm resources nursery; and on BEC108A from Malipo, Wenshan, Yunnan coffee planting base.

*Colletotrichum karstii* Y.L. Yang, Zuo Y. Liu, K.D. Hyde and L. Cai, 2012 [42].

**Figure 6:**
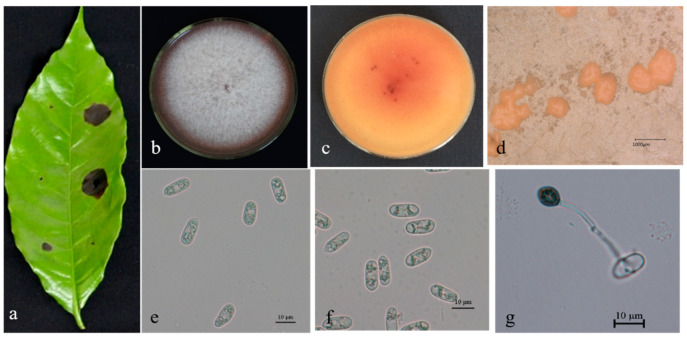
*Colletotrichum karstii* (Bai1) on leave of host plant (**a**); surface (**b**) and reverse (**c**) sides of colony after incubation for 5 days on PDA; (**d**) conidiomata; (**e**,**f**) conidia; (**g**) conidial appressoria. Scale bars: 10 μm (**e**–**g**).

Description. On PDA. Colonies raised, white-to-gray, dense aerial mycelium; reverse: red, cottony surface of colony with numerous small orange/apricot conidiomata; colonies 40–52 mm in diameter after 5 days at 25 °C. Vegetative hyphae hyaline-to-medium-brown, usually smooth-walled, septate, and branched; chlamydospores and setae not observed. Conidiophores are either directly formed from hyphae or from cushion of spherical, hyaline cells, septate and sometimes branched. Conidiogenous cells are hyaline-to-pale-brown, cylindrical, and straight-to-flexuous. Conidia are hyaline, usually aseptate, smooth-walled, and ovoid-to-cylindrical or clavate, with both ends rounded, or one end rounded and one end acute, and are guttulate, granular; (10.5–) 12.1–13.5 (–17.5) × 4.0–5.5 μm, mean ± SD = 13.4 ± 1.7 × 4.5 ± 0.4 μm, L/W ratio = 2.2. Appressoria formed in slide culture: (6.9–) 8.8-8.1 (–9.5) × 8.1–10.0 μm, mean ± SD = 11.6 ± 1.0 × 8.9 ± 0.6 μm, L/W ratio = 1.0, formed from branched mycelia, terminal, brown-to-dark-brown, variable in shape, and irregular. Sexual state not observed in culture (Figure 6).

Specimens examined. Diseased leaves on cultures Bai1, Bai2, Bai4, and Bai5 from Hainan, Baisha coffee planting base; on cultures HG11, HG12, and HG14 from Yunnan, Mangshi, Hougu coffee base; on cultures BEC26C, BEC32A, BEC33B, BEC38A, and RBEC151 from Yunnan tropical crop institute demonstration base; and on cultures BEC52A, BEC107A, and BEC117B from Malipo, Wenshan, Yunnan coffee planting base.

*Colletotrichum cliviicola* Damm and Crous, 2019, nom. nov. [43].

**Figure 7:**
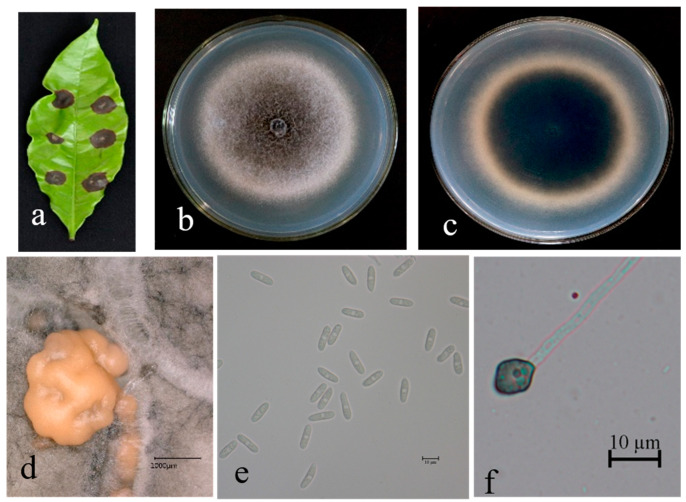
*Colletotrichum cliviicola* (FS4-2) on leaf of host plant (**a**); surface (**b**) and reverse (**c**) sides of colony after incubation for 5 days on PDA; (**d**) conidiomata; (**e**) conidia; (**f**) conidial appressoria. Scale bars: 10 μm (**e**,**f**).

Description. On PDA. Colonies 58–65 mm in diameter after 5 days at 25 °C. Edge is flat; aetial mycelium is dense, cottony, and gray-to-dark-gray at center; reverse is grayish-green with white halo. Conidiomata are apricot/orange; vegetative hyphae are hyaline-to-medium-brown, usually smooth-walled, septate, and branched. Chlamydospores and setae not observed. Conidiophores are either directly formed from hyphae or from cushion of spherical, hyaline cells, septate and sometimes branched. Conidiogenous cells are hyaline-to-pale-brown, cylindrical, and straight-to-flexuous. Conidia are hyaline and usually aseptate, sometimes becoming 1–2 septate with age; are smooth-walled and ovoid-to-cylindrical or clavate, with both ends rounded, or one end rounded and one end acute; and are guttulate and granular; (13.9–) 15.2–20.1 (–23.4) × 4.8–5.6 μm, mean ± SD = 17.7 ± 1.0 × 5.2 ± 0.7 μm, L/W ratio = 3.4. Appressoria formed in slide culture: (5.2–) 6.2–11.3 (–13.8) × 5.8–9.6 μm, mean ± SD = 8.7 ± 0.5 × 7.5 ± 0.9 μm, L/W ratio = 1.2, formed from branched mycelia, terminal, brown-to-dark-brown, variable in shape, and irregular. Sexual state not observed in culture (Figure 7).

Specimens examined. Diseased leaves on culture FS4-2 from Hainan, Fushan coffee planting base.

*Colletotrichum brevisporum* Phoulivong, Noireung, L. Cai and K.D. Hyde 2012 [44].

**Figure 8:**
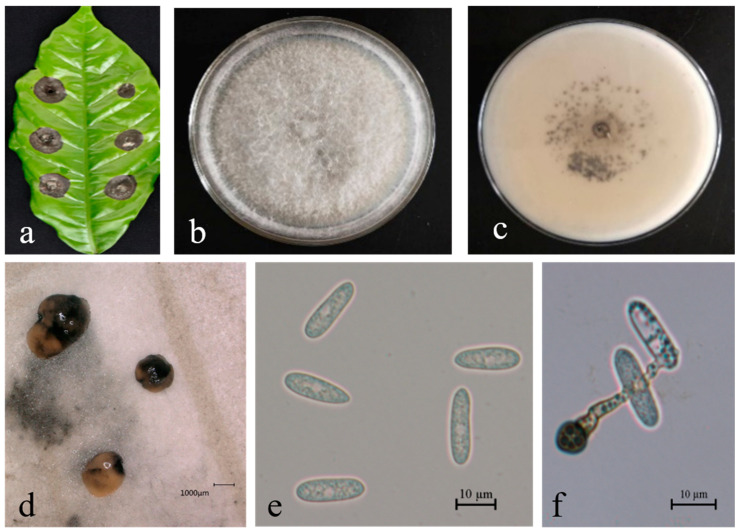
*Colletotrichum brevisporum* (BEC92) leaf of host plant (**a**); surface (**b**) and reverse (**c**) sides of colony after incubation for 5 days on PDA; (**d**) conidiomata; (**e**) conidia; (**f**) conidial appressoria. Scale bars: 10 μm (**e**,**f**).

Description. On PDA. Colonies raised, white-to-gray, with dense aerial mycelium; reverse is white; conidiomata are apricot and black. Colonies 58–67 mm in diameter after 5 days at 25 °C. Vegetative hyphae are hyaline-to-medium brown, usually smooth-walled, septate, and branched; chlamydospores and setae not observed. Conidiophores either directly formed from hyphae or from cushion of spherical, hyaline cells, septate and sometimes branched. Conidiogenous cells are hyaline-to-pale-brown, cylindrical, and straight-to-flexuous. Conidia are hyaline and usually aseptate, sometimes becoming 1–2 septate with age; are smooth-walled and ovoid-to-cylindrical or clavate, with both ends rounded, or one end rounded and one end acute; and are guttulate and granular; (13.4–) 17.0–19.5 (−21.7) × 4.8–6.1 μm, mean ± SD = 18.3 ± 1.2 × 5.6 ± 0.8 μm, L/W ratio = 3.3. Appressoria formed in slide culture: (6.5–) 7.5–14.3 (−15.3) × 6.0–11.0 μm, mean ± SD = 10.8 ± 1.1 × 8.2 ± 1.0 μm, L/W ratio = 1.1, formed from branched mycelia, terminal, brown-to-dark-brown, variable in shape, and irregular. Sexual state not observed in culture (Figure 8).

Specimens examined. Diseased leaves on culture BSC15-2 from Hainan, Baisha coffee planting base; and on BEC92 from Yunnan agricultural germplasm resources nursery.

*Colletotrichum gigasporum* E.F. Rakotoniriana and Munaut, 2013 [45].

**Figure 9:**
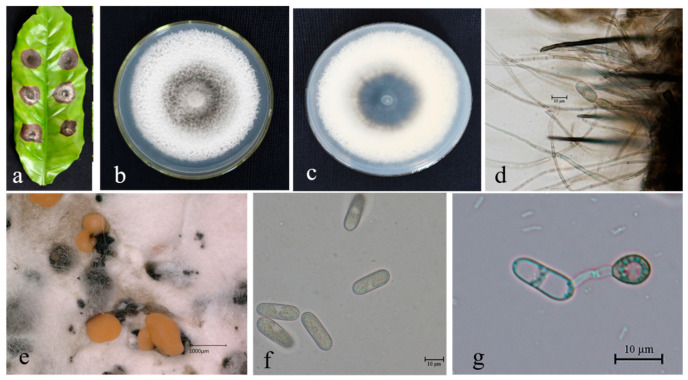
*Colletotrichum gigasporum* (BEC191A) on leaf of host plant (**a**); surface (**b**) and reverse (**c**) sides of colony after incubation for 7 days on PDA; (**d**) conidiophore, conidiogenous cells, and conidia; (**e**) conidiomata; (**f**) conidia; (**g**) conidial appressoria. Scale bars: 10 μm (**f**,**g**).

Description. On PDA. Colonies 42–56 mm in diameter after 7 days at 25 °C. Aerial mycelium are white and cottony; surface of colony has numerous small orange conidiomata; reverse is white at first, then gray-to-pale-olive at center. Conidia are hyaline, aseptate, smooth-walled, and ovoid-to-cylindrical, with both ends bluntly round; (17.3–) 22.2–28.5 (−31.8) × 8.2–9.8 μm, mean ± SD = 25.7 ± 1.1 × 8.9 ± 0.5 μm, L/W ratio = 2.7. Appressoria formed in slide culture: (8.8–) 10.0–19.1 (−21.3) × 6.5–11.3 μm, mean ± SD = 13.5 ± 1.0 × 8.9 ± 0.8 μm, L/W ratio = 1.2, formed from branched mycelia, terminal, brown-to-dark-brown, variable in shape, and irregular. Sexual state not observed in culture (Figure 9).

Specimen examined. Diseased leaves on culture BEC191A from Yunnan, Xinghua farm.

### 3.3. Species Diversity of Colletotrichum in China

**Figure 10:**
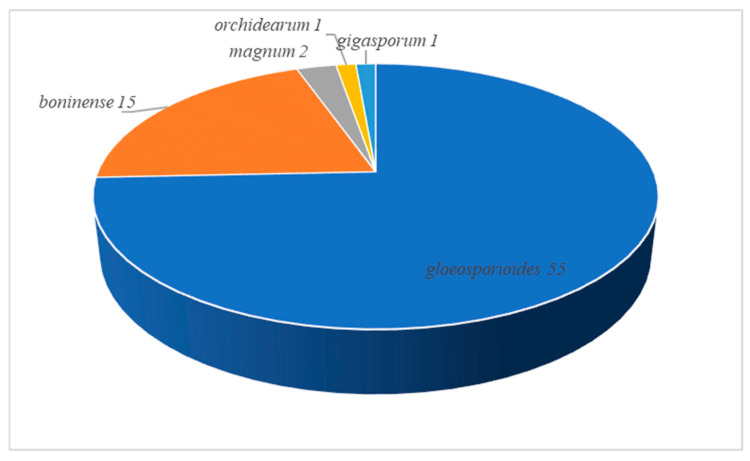
Statistics of *Colletotrichum* species complexes in this study.

**Figure 11:**
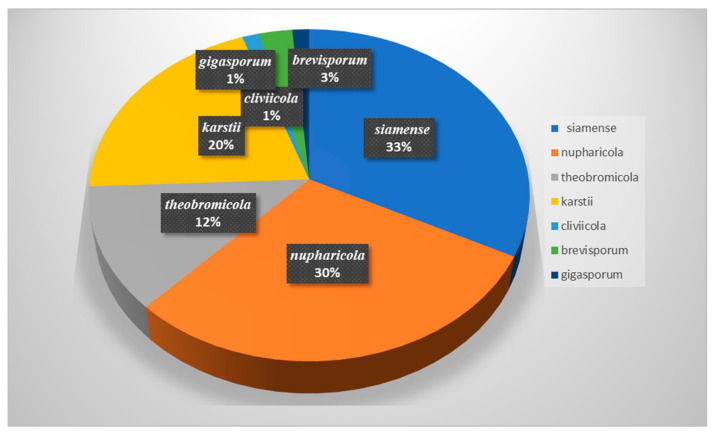
Statistics of *Colletotrichum* species in this study.

Based on BLASTn searches and phylogenetic analyses of single and multi-locus sequences, 74 strains were assigned to seven species, belonging to five species complexes. The majority of the analyzed strains belonged to the *C. gloeosporioides* species complex, followed by the *C. boninense* species complex, the *C. magnum* species complex, the *C.* orchidearum species complex, and the *C*. gigasporum species complex (Figure 10). Among all the identified species, *C. siamense* was the most common taxon, accounting for 32.43% of the total isolates obtained, followed by *C. nupharicola*, accounting for 29.73%, and *C. theobromicola*, accounting for 12.16%. *C. karstii* accounted for 20.27%, while *C. cliviicola*, *C. brevisporum*, and *C. gigasporum* had an incidence of <5% (Figure 11).

### 3.4. Pathogenicity Assay

Pathogenicity was tested on detached coffee leaves in vitro for two *C. brevisporum* (BSC92, BSC15-2), *C. cliviicola* (FS4-2), one isolate from *C. gigasporum* (BEC191A), *C. karstii* (Bai 1, BEC26C), two *C. nupharicola* (BSC1-2, BEC106B), two *C. siamense* (BSC4-1, HG 9), and *C. theobromicola* (BSC1-3, IG3) At 7 dpi, all the tested isolates were pathogenic to leaves of arabica; the incidence of infection ranged from 8.5 to 100%. All isolates except for BEC92 (*C. brevisporum*) and IG3 (*C. theobromicola*) were pathogenic to robusta, with the incidence of infection ranging from 33.3 to 100%. The incidence of infection of both young and mature leaves varied among species/isolates (Table 3).

## 4. Discussion

Effective management of plant diseases relies on a comprehensive understanding of the host, pathogen, and environmental factors. However, in the case of *Colletotrichum* spp., each of these aspects is highly complex and multi-dimensional. Accurate identification of the pathogen is crucial for developing appropriate and effective disease management strategies. This study represents the first effort to utilize a polyphasic approach to characterize *Colletotrichum* spp. associated with coffee anthracnose in China.

The polyphasic approach, which integrates phenotypic characteristics and multi-locus phylogeny, has previously been successfully employed to resolve taxonomic complexities within the *Colletotrichum* genus. This method has proven effective in clarifying species delineations and addressing challenges related to the identification and classification of *Colletotrichum* species [20,25,46]. In this study, a thorough phenotypic and molecular analysis of 74 isolates revealed that seven species of *Colletotrichum* are associated as causal agents of coffee anthracnose in China. Specifically, *C. siamense*, *C. nupharicola*, and *C. theobromicol*, belonging to the *C. gloeosporioides* species complex, and *C. karstii*, *C. cliviicola*, *C. brevisporum*, and *C. gigasporum*. This finding highlights the diversity and complexity of *Colletotrichum* species involved in coffee anthracnose pathology. Among the species complexes, the *C. gloeosporioides* species complexes contain more species than the other species complexes. Nearly 75% of all the isolates obtained from the coffee plantations samples were from the *C. gloeosporioides* species complex. The majority of *Colletotrichum* isolates associated with coffee anthracnose in Vietnam belonged to the *C. gloeosporioides* species complex [21]. Studies also indicate that the *C. gloeosporioides* species complex is the dominant species complex in rubber trees and coffee trees in Hainan [26]. The composition of *Colletotrichum* species differ between different regions and coffee varieties; a large-scale survey is needed to confirm this differential distribution.

*C. siamense* is reported to originate from coffee berries in Thailand [22], and has a wide range of hosts and geographical distributions. *C. siamense* is reported to infect plants in Australia and Mexico [27,47]. A study found that most isolates from the *C. gloeosporioides* species complex that caused leaf anthracnose of rubber trees in Hainan were *C. siamense* [26]. In this study, 32.43% of isolates from coffee plants were identified to be this species.

Although morphological characteristics alone are insufficient to distinguish *Colletotrichum* species at the individual species level, they remain important taxonomic tools for identifying species within broader species complexes [16]. For instance, conidia of species in the *C. gigasporum* complex are notably larger than those of other species complexes [13], while typical conidia of the *C. boninense* complex are cylindrical, with a prominent basal scar [32]. However, many species across various complexes, such as the *C. dracaenophilum*, *C. magnum*, and *C. orchidearum* species complexes, as well as singleton species, produce cylindrical conidia with rounded ends, a feature commonly associated with the *C. gloeosporioides* complex [43]. In general, species within the *C. acutatum*, *C. bambusicola*, *C. boninense*, *C. dracaenophilum*, *C. gigasporum*, *C. gloeosporioides*, *C. magnum*, *C. orbiculare*, and *C. orchidearum* species complexes produce straight conidia. In contrast, species in the *C. caudatum*, *C. dematium*, *C. graminicola*, *C. spaethianum*, and *C. truncatum* complexes produce curved conidia [17,20,30,32,33,34,43,48,49,50,51,52,53]. Notably, species complexes with curved conidia are distributed throughout the phylogenetic tree, suggesting that the evolution of curved spores may have occurred multiple times independently within the genus. This highlights the complexity and diversity of morphological traits in the *Colletotrichum* taxonomy.

Many *Colletotrichum* species lack consistent and reliable diagnostic morphological features, making their identification challenging [25]. As a result, molecular methods were employed in this study to accurately identify *Colletotrichum* species. Both single-locus ApMat and multi-locus sequences (ITS, *ACT*, *CHS-1*, GAPDH) were utilized for phylogenetic analysis. The phylogenetic trees generated from both approaches yielded similar results, demonstrating that the *ApMat* marker provides superior phylogenetic resolution compared to other loci, and is capable of differentiating most species within the *C. gloeosporioides* species complex. These findings align with previous studies by Silva et al. [24] and Vieira et al. [54]. The *ApMat* marker shows potential for development as a tool for the rapid diagnosis of species within the *C. gloeosporioides* complex associated with anthracnose on coffee in China. This could significantly enhance the efficiency and accuracy of species identification in the future.

Research on the relationships between hosts and the pathogenicity of pathogens provides an important theoretical foundation for the control of plant diseases. By uncovering the molecular mechanisms of host–pathogen interactions, more precise and sustainable control strategies can be developed, such as disease-resistant breeding, biological control, and gene-editing technologies [55,56]. In this study, pathogenicity assay confirmed that all tested isolates of *Colletotrichum* species were pathogenic to *Arabica* coffee leaves, causing infections with varying degrees of severity. The incidence of infection ranged from 8.5% to 100%, indicating significant variability in the ability of different isolates to infect arabica leaves. Most isolates were also pathogenic to *Robusta* coffee leaves, except for two isolates: BSC92 (*C. brevisporum*) and IG3 (*C. theobromicola*). The incidence of infection on *Robusta* leaves ranged from 33.3% to 100%, suggesting that *Robusta* may exhibit some level of resistance or reduced susceptibility to certain isolates compared to *Arabica*. The study revealed significant differences in pathogenicity among the *Colletotrichum* species or isolates; *C. siamense* (isolates BSC4-1 and HG 9) and *C. karstii* (isolates Bai 1 and BEC26C) showed high pathogenicity, with infection incidences reaching up to 100% on both *Arabica* and *Robusta* leaves. *C. brevisporum* (isolate BSC92) and *C. theobromicola* (isolate IG3) exhibited lower or no pathogenicity on *Robusta* leaves, suggesting potential host-specific interactions or reduced virulence in these isolates. Even within the same species, isolates demonstrated variability in their ability to infect coffee leaves. For instance, *C. nupharicola* isolates (BSC1-2 and BEC106B) showed differing infection incidences, highlighting the importance of genetic or phenotypic variation among isolates. The high pathogenicity of certain isolates, such as those from *C. siamense* and *C. karstii*, underscores the need for targeted disease management strategies, particularly for *Arabica* coffee, which is more susceptible. The reduced susceptibility of *Robusta* coffee to some isolates (e.g., BSC92 and IG3) suggests potential genetic resistance traits that could be explored for breeding programs to develop more resistant coffee varieties. Variability in pathogenicity among isolates and species highlights the importance of monitoring *Colletotrichum* populations in coffee plantations, as shifts in species or isolate composition could impact disease severity and spread.

## 5. Conclusions

In this study, five *Colletotrichum* species were obtained from coffee leaves with anthracnose symptoms in China. Two species were reported to be associated with coffee for the first time. It is also encouraging that *C. kahawae*, the main pathogen threatening coffee production in Africa, has not been observed in China. The results of this study can be valuable for developing sustained management strategies for anthracnose of coffee. Further large-scale surveys and pathogenicity testing are necessary before effective disease management strategies can be formulated and implemented.

## Figures and Tables

**Table 1:** Sampling location of night coffee plantation in Yunan and Hainan, China.

Location	Host Plant	GPS Coordinates	No. of Samples	No. of Isolates
Yunnan tropical crop institute demonstration base	Arabica	22°47′47″; 100°58′59″	12	9
Nandao River, Yunnan province coffee planting base	Arabica	22°36′52″; 101°0′38″	9	6
Malipo, Wenshan, Yunnan coffee planting base	Arabica	23°12′47″; 104°54′1″	10	7
Yunnan agricultural germplasm resources nursery	Arabica	22°37′37″; 100°59′47″	17	13
Yunnan, Xinghua farm	Arabica	24°11′41″; 98°10′11″	4	2
Yunnan, Mangshi, Bandong coffee planting base	Arabica	24°15′7″; 98°7′28″	16	3
Yunnan, Mangshi, Hougu coffee base	Arabica	24°21′38″; 98°27′44″	10	8
Hainan, Fushan coffee planting base	Robusta/Arabica	19°49′55″; 109°55′33″	8	4
Hainan, Baisha coffee planting base	Robusta/Arabica	19°9′55″; 109°28′39″	34	22

**Table 2:** Gene regions and PCR primers used in this study.

Locus	Gene	Primers	Sequence (5′-3′)	Reference
Internal transcribed spacer regions with intervening 5.8S nrRNA gene	*ITS*	ITS1 ITS4	TCCGTAGGTGAACCTGCGG TCCTCCGCTTATTGATATGC	White et al., 1990 [36]
Partial actin gene	*ACT*	ACT-512F ACT-783R	ATGTGCAAGGCCGGTTTCGC TACGAGTCCTTCTGGCCCAT	Carbone et al., 1999 [37]
Partial chitin synthase 1 gene	*CHS-1*	CHS-79F CHS-354R	TGGGGCAAGGATGCTTGGTTGAAG TGGAAGAACCATCTGTGAGAGTTG	Carbone et al., 1999 [37]
Partial glyceraldehyde-3-phosphate dehydrogenase gene	*GAPDH*	GDF GDR	GCCGTCAACGACCCCTTCATTGA GGGTGGAGTCGTACTTGAGCATGT	Templeton et al., 1992 [38]
Partial mating type protein 1-2-1 gene	*ApMat*	AMF AMR	TCATTCTACGTATGTGCCCG CCAGAAATACACCGAACTTGC	Silva et al., 2012 [24]

**Table 3:** The incidence of infection of *Colletotrichum* spp. inoculated on leaves of arabica and robusta ^a^.

Species	Isolates	Variety	Arabica		Robusta	
			Young Leaf	Mature Leaf	Young Leaf	Mature Leaf
*C. brevisporum*	BEC92	Arabica	16.7	8.5	0	0
	BSC15-2	Robusta	33.3	25	50	33.3
*C. cliviicola*	FS4-2	Robusta	25	16.7	50	16.7
*C. gigasporum*	BEC191A	Arabica	75	66.7	50	50
*C. karstii*	Bai1	Robusta	83.3	75	100	66.7
	BEC26C	Arabica	33.3	16.7	50	33.3
*C. nupharicola*	BSC1-2	Robusta	100	50	75	75
	BEC106B	Arabica	33.3	33.3	50	33.3
*C. siamense*	BSC4-1	Robusta	50	75	75	100
	HG 9	Arabica	75	50	50	50
*C. theobromicola*	BSC1-3	Robusta	75	100	100	100
	IG3	Arabica	33.3	16.7	0	0

^a^ infection incidence (%) = (number of infected points on leaves/number of inoculated points on leaves) × 100; twelve inoculation points for each leaf.

## Data Availability

The original contributions presented in this study are included in the article. Further inquiries can be directed to the corresponding author.

## Supplementary Materials

The following supporting information can be downloaded at https://www.mdpi.com/article/10.3390/microorganisms13030512/s1. Tables S1 and S2. Species and GenBank accession numbers of DNA sequences used in this study with new sequences in bold.
