# Supplementary material for: Diversity and Pathogenicity of Colletotrichum Species Causing Coffee Anthracnose in China
Source: Microorganisms. 2025 Feb 26;13(3):512. doi: 10.3390/microorganisms13030512 (PMC11946238; doi:10.3390/microorganisms13030512)
Supplement: Supplementary file 1 [file microorganisms-13-00512-s001.zip › Table S1 .pdf]

Table S1 Species and GenBank accession numbers of DNA sequences used in this study with new sequences in bold

Species	Strain/Isolate	Host/Substrate	*ApMat* GenBank accession number
C. aenigma	IY1059	*Persea americana*	KM360143.1
C. aeschynomene	ICMP 17673	*Aeschynomene virginica*	KM360145
C. alatae	CBS 304	*Dioscorea alata*	KC888932
C. alienum	ICMP 12071	*Malus domestica*	KM360144
C. artocarpicola	3152	*Artocarpus heterophyllus*	MK344199
C. asianum	n04	*Coffea arabica*	LC777796
C. boninens	CBS 123755	*Crinum asiaticum* var. sinicum	KU375659
C. camelliae	CMP 10643	*Camellia × williams*	KJ954625
C. chrysophilum	CMM4268	*Musa* sp.	OR001712
C. cigarro	ICMP 19122	*Vaccinium* sp.	MH346085
C. clidemiae	ICMP 18658	Clidemia hirta	KC888929
C. conoides	CAUG17	*Capsicum annuum*	MG198007
C. endophytica	CAUG28	*Capsicum annuum*	KU251735
C. fructicola	ICMP:18646	*Coffea arabica*	JQ807838
C. gloeosporioides	MTCC 10323	*Citrus sinensi*	JQ807843
C. grevilleae	CBS 132879	*Grevillea* sp.	JQ894578
C. hebeiense	JZB330028	*Vitis vinifera*	KF377573
C. horii	ICMP 10492	Diospyros kaki	JQ807840
C. hymenocallidis	MTCC10286	*Malus domestica*	JQ807842
C. hystricis	CPC 28153	*Citrus hystrix*	KJ954561
C..javanense	CBS144963	-----	KR134301
C. jiangxiense	LF687	*Camellia sinensis*	KJ954561
C. kahawae	IMI 319418=ICMP 17816	*Coffea arabica*	MH346047

C. makassarense	CBS 143664=CPC 28612	*Capsicum annuum*	MH728831
C. musae	CBS 116870=ICMP 19119	*Musa* sp.	OR371741
C. nupharicola	CBS 470.96=ICMP 18187	N*uphar lutea* subsp. polysepala	JX145319
C. noveboracense	AFK289	*Apple*	MN622870
C. perseae	GA100	*Persea americana*	PQ227313
C. psidii	ICMP 19120	*Psidium* sp.	KC888931
C. queenslandicum	ICMP 1778	*Carica papaya*	KC888928
C. salsolae	ICMP 19051	*Salsola tragus*	KC888925
C. siamense	AGMy0249	*Coffea arabica*	MT028344
C. tainanense	CBS 143666	*Capsicum annuum*	MH728836
C. theobromicola	MTCC11350	*Theobroma cacao*	KC790726
C. ti	ICMP 4832	*Cordyline* sp.	KM360146
C. tropicale	C08	*Theobroma cacao*	LC777793
C. viniferum	GZAAS58601	*Vitis vinifera*	PQ554000
C. xanthorrhoeae	BRIP 45094*=ICMP 17903 = CBS	*Xanthorrhoea preissii*	KC790689.1
	**BSC10-2**	***Coffea Robusta***	**C_AA078276.1**
	**BEC41B**	***Coffea arabica***	**C_AA072800.1**
	**BSC9-1**	***Coffea Robusta***	**C_AA072811.1**
	**BEC70A**	***Coffea arabica***	**C_AA072822.1**
	**RBEC193B**	***Coffea arabica***	**C_AA072833.1**
	**BEC176B**	***Coffea arabica***	**C_AA072844.1**
	**HG 9**	***Coffea arabica***	**C_AA072850.1**
	**HG 6**	***Coffea arabica***	**C_AA072851.1**
	**BEC59A**	***Coffea arabica***	**C_AA072852.1**
	**CF2**	***Coffea Robusta***	**C_AA072853.1**
	**BSC4-1**	***Coffea Robusta***	**C_AA072801.1**

**BSC7-2**	***Coffea Robusta***	**C_AA072802.1**
**BSC2-2**	***Coffea Robusta***	**C_AA072803.1**
**BD 18**	***Coffea arabica***	**C_AA072804.1**
**BSC9-3**	***Coffea Robusta***	**C_AA072805.1**
**F3-1**	***Coffea Robusta***	**C_AA072806.1**
**BEC77D**	***Coffea arabica***	**C_AA072807.1**
**BEC76B**	***Coffea arabica***	**C_AA072808.1**
**BSC7-1**	***Coffea Robusta***	**C_AA072809.1**
**BEC91B**	***Coffea arabica***	**C_AA072810.1**
**BEC75A**	***Coffea arabica***	**C_AA072812.1**
**BD 23**	***Coffea arabica***	**C_AA072813.1**
**BD 15**	***Coffea arabica***	**C_AA072814.1**
**BSC2-1**	***Coffea Robusta***	**C_AA072815.1**
**BSC14-2**	***Coffea Robusta***	**C_AA072816.1**
**IG3**	***Coffea Robusta***	**C_AA072817.1**
**BSC13-1**	***Coffea Robusta***	**C_AA072818.1**
**BSC8-3**	***Coffea Robusta***	**C_AA072819.1**
**BSC8-1**	***Coffea Robusta***	**C_AA072820.1**
**BSC6-1**	***Coffea Robusta***	**C_AA072821.1**
**BSC1-3**	***Coffea Robusta***	**C_AA072823.1**
**RL 32**	***Coffea arabica***	**C_AA072824.1**
**BEC108A**	***Coffea arabica***	**C_AA072825.1**
**BEC106B**	***Coffea arabica***	**C_AA072826.1**
**BEC126B**	***Coffea arabica***	**C_AA072827.1**
**BSC14-1**	***Coffea Robusta***	**C_AA072828.1**
**HG 7**	***Coffea arabica***	**C_AA072829.1**

**HG 8**	***Coffea arabica***	**C_AA072830.1**
**RL 24**	***Coffea arabica***	**C_AA072831.1**
**RL 31**	***Coffea arabica***	**C_AA072832.1**
**RL 33**	***Coffea arabica***	**C_AA072834.1**
**BSC15-1**	***Coffea Robusta***	**C_AA072835.1**
**BSC1-2**	***Coffea Robusta***	**C_AA072836.1**
**RL 30**	***Coffea arabica***	**C_AA072837.1**
**RL 29**	***Coffea arabica***	**C_AA072838.1**
**RL 28**	***Coffea arabica***	**C_AA072839.1**
**RL 25**	***Coffea arabica***	**C_AA072840.1**
**HG 10**	***Coffea arabica***	**C_AA072841.1**
**BEC156A**	***Coffea arabica***	**C_AA072842.1**
**BEC80A**	***Coffea arabica***	**C_AA072843.1**
**BEC77A**	***Coffea arabica***	**C_AA072845.1**
**BEC127B**	***Coffea arabica***	**C_AA072846.1**
**BEC14B**	***Coffea arabica***	**C_AA072847.1**
**BEC76A**	***Coffea arabica***	**C_AA072848.1**
**BEC10A**	***Coffea arabica***	**C_AA072849.1**
