# Supplementary material for: Diversity and Pathogenicity of Colletotrichum Species Causing Coffee Anthracnose in China
Source: Microorganisms. 2025 Feb 26;13(3):512. doi: 10.3390/microorganisms13030512 (PMC11946238; doi:10.3390/microorganisms13030512)
Supplement: Supplementary file 1 [file microorganisms-13-00512-s001.zip › Table S2.pdf]

Table S2 Species and GenBank accession numbers of DNA sequences used in this study with new sequences in bold

Species	Strain/Isolate	Host/Substrate	GenBank accession number			
			ITS	ACT	CHS-1	GAPDH
Colletotrichum boninense species complex						
C. annellatum	CBS:129826	*Hevea brasiliensis*	JQ005222.1	JQ005570.1	JQ005396.1	JQ005309.1
C. beeveri	CBS:128527	*Brachyglottis repanda*	MH865007.1	JQ005519.1	JQ005345.1	JQ005258.1
C. boninense	CBS 123755	*Crinum asiaticum var. sinicum*	JQ005153	JQ005501	JQ005327	JQ005240
C. brasiliense	CBS:128501	*Passiflora edulis f. flavicarpa,*	MH864997.1	JQ005583.1	JQ005409.1	JQ005322.1
C. brassicicola	CBS:101059	*Brassica oleracea var. gemmifera*	JQ005172.1	JQ005520	JQ005346.1	JQ005259.1
C. bromeliacearum	LC13855	---	MZ595835	MZ664132.1	MZ799269.1	MZ664079.1
C. camelliae-japonicae	LC6415	----	KX853164.1	KX893575.1	OQ683324.1	OQ683321.1
C. catinaense	CPC 28149	*Citrus reticulata*	KY856401.1	KY855972.1	KY856137.1	KY856225.1
C. celtidis	GUCC 12023	*Plum*	OP723046.1	MW151580.1	OM655125.1	OM655151.1
C. chamaedoreae	ZHKUCC 23-0856	*Dypsis lutescens*	OR272088.1	OR493811.1	OR493839.1	OR493867.1
C. citricola	ZHKUCC 24-0045	*Citrus unshiu*	PP434621.1	PP444986.1	PP465752.1	PP465785.1
C. colombiense	CBS:129818	*Passiflora edulis*	MH865416.1	JQ005522.1	JQ005348.1	JQ005261.1
C. condaoense	CBS:134299	*Ipomoea pes-caprae*	MH229914.1	-----	MH229926.1	MH229920.1
C. constrictum	CBS:128504	*Citrus limon*	JQ005238.1	JQ005585.1	JQ005412.1	JQ005325.1
C. cymbidiicola	JCSH C1-3	*Cymbidium sichuanicum*	PP982545.1	PP965567.1	OR493837.1	OL801342.1
C. dacrycarpi	CBS:130241	*Dacrycarpus dacrydioides*	MH865628.1	JQ005584.1	JQ005410.1	JQ005323.1
C. diversum	LC11292	*Philodendron selloum*	MZ595844.1	MZ664142.1	MZ799272.1	MZ664081.1
C. doitungense	MFLUCC 14_0128	*Dendrobium* sp.	MF448524.1	MH376385.1	------	MH376385.1
C. feijoicola	CPC:34245	*Acca sellowiana* (Feijoa)	MK876414.1	MK876465.1	MK876471.1	MK876474.1
C. fici	NCYUCC 19-0337	*Ficus ampelas*	MW114366.1	MW151584.1	MW177700.1	------
C. hippeastri	CBS:125376	*Hippeastrum vittatum*	MH863510.1	JX009485.1	GQ856725.1	JQ005319.1

C. karsti	2584	*Vanda* sp.	OK360947.1	OK040201.1	OK040210.1	OK040215.1
C. karstii	CBS:113087	*Malus* sp	JQ005181.1	JQ005529.1	JQ005355.1	JQ005268.1
	**BEC107A**	***Coffea arabica***	**PP838104**	**C_AA076825.1**	**C_AA076973.1**	**C_AA077047.1**
	**Bai4**	***Coffea Robusta***	**PP838105**	**C_AA076827.1**	**C_AA076975.1**	**C_AA077049.1**
	**BEC32A**	***Coffea arabica***	**PP838100**	**C_AA076821.1**	**C_AA076969.1**	**C_AA077043.1**
	**BEC33B**	***Coffea arabica***	**PP838099**	**C_AA076820.1**	**C_AA076968.1**	**C_AA077042.1**
	**RBEC151**	***Coffea arabica***	**PP838098**	**C_AA076819.1**	**C_AA076967.1**	**C_AA077041.1**
	**BEC38A**	***Coffea arabica***	**PP838097**	**C_AA076818.1**	**C_AA076966.1**	**C_AA077040.1**
	**HG12**	***Coffea arabica***	**PP838091**	**C_AA076811.1**	**C_AA076959.1**	**C_AA077033.1**
	**BEC117B**	***Coffea arabica***	**PP838102**	**C_AA076823.1**	**C_AA076971.1**	**C_AA077045.1**
	**HG14**	***Coffea arabica***	**PP838092**	**C_AA076812.1**	**C_AA076960.1**	**C_AA077034.1**
	**Bai2**	***Coffea arabica***	**PP838093**	**C_AA076813.1**	**C_AA076961.1**	**C_AA077035.1**
	**Bai1**	***Coffea Robusta***	**PP838094**	**C_AA076814.1**	**C_AA076962.1**	**C_AA077036.1**
	**HG11**	***Coffea arabica***	**PP838095**	**C_AA076816.1**	**C_AA076964.1**	**C_AA077038.1**
	**BEC26C**	***Coffea arabica***	**PP838096**	**C_AA076817.1**	**C_AA076965.1**	**C_AA077039.1**
	**Bai5**	***Coffea Robusta***	**PP838101**	**C_AA076822.1**	**C_AA076970.1**	**C_AA077044.1**
	**BEC52A**	***Coffea arabica***	**PP838103**	**C_AA076824.1**	**C_AA076972.1**	**C_AA077046.1**
C. limonicola	CPC 27862	*Citrus limon*	KY856473.1	KY856046.1	KY856214.1	KY856297.1
C. novae-zelandiae	CBS:128505	*Capsicum annuum*	MH864998.1	JQ005576.1	JQ005402.1	JQ005315.1
C. oncidii	OORC24	*Oncidium* sp.	KU239205.1	KU239884.1	KU239428.1	KU239666.1
C. parsonsiae	CBS:128525	*Parsonsia capsularis*	MH865006.1	JQ005581.1	JQ005407.1	JQ005320.1
C. petchii	CBS:378.94	*Dracaena marginata*	JQ005224.1	JQ005571.1	JQ005397.1	JQ005310.1
C. phyllanthi	CBS:175.67	*Phyllanthus acidus*	JQ005221.1	JQ005569.1	JQ005395.1	JQ005308.1
C. torulosum	CBS:128544	*Solanum melongena*	MH865015.1	Q005512.1	JQ005338.1	JQ005251.1
C. watphraense	MFLUCC 14_0123	*Dendrobium* sp.	MF448523.1	MH376384.1	-----	MH049479.1
Colletotrichum magnum species complex:						

C. brevisporum	CP-H1	*Papaya Maradol*	OK160666.1	OK169930.1	OK169931.1	OK169932.1
	**BSC15-2**	***Coffea Robusta***	**PP838106**	**C_AA076828.1**	**C_AA076976.1**	**C_AA077050.1**
	**BEC92**	***Coffea arabica***	**PP838107**	**C_AA076829.1**	**C_AA076977.1**	**C_AA077051.1**
C. cacao	CBS:119297	*Theobroma cacao*	MG600772.1	MG600976.1	MG600878.1	MG600832.1
C. guangdongense	TL10-1	*Passion*	PP690782.1	PQ007589.1	PQ259886.1	PP977495.1
C. liaoningense	CAUOS2	*chili pepper*	KP890104.1	KP890097.1	KP890127.1	KP890135.1
C. lobatum	IMI79736	*Piper marginatum f. catalpifolium*	MG600768.1	MG600972.1	MG600874.1	MG600828.1
C. magnum	TZ02	*Citrullus lanatus*	OP740803.1	LC797856.1	LC797816.1	LC797736.1
C. merremiae	CBS:124955	*Merremia umbellata*	MG600765.1	MG600969.1	MG600872.1	MG600825.1
C. okinawense	MAFF240517	*petiole of Carica papaya*	MG600767.1	MG600971.1	-----	MG600827.1
C. panamense	CBS:125386	*Merremia umbellata*	MG600766.1	MG600970.1	MG600873.1	MG600826.1
Colletotrichum orchidearum species complex						
C. cattleyicola	Col-052	*Cattleya* sp	OR515650.1	OR636335.1	OR636351.1	OR636327.1
C. cliviicola	CBS:125375	*Clivia miniata*	MH863509.1	MG600939.1	JX519232.1	JX546611.1
	**FS4-2**	***Coffea Robusta***	**PP838108**	**C_AA076830.1**	**C_AA076978.1**	**C_AA077052.1**
C. monsterae	LC13871	*Monstera deliciosa*	MZ595897.1	MZ664195.1	MZ799351.1	MZ664121.1
C. musicola	CBS:132885	*Musa* sp.	MG600736.1	MG600942.1	MG600853.1	MG600798.1
C. orchidearum	CBS:135131	*Dendrobium nobile*	MG600738.1	MG600944.1	MG600855.1	MG600800.1
C. piperis	IMI71397	*Piper nigrum*	MG600760.1	MG600964.1	MG600867.1	MG600820.1
C. plurivorum	CBS:125474	*Coffee*	MG600718.1	MG600925.1	MG600841.1	MG600781.1
C. reniforme	LC8230	*Smilax cocculoides*	MZ595847.1	MZ664145.1	MZ799290.1	MZ664110.1
C. sojae	ATCC_ 62257	*Glycine max*	MG600749.1	MG600954.1	MG600860.1	MG600810.1
C. subplurivorum	CNUCC_ 833B-1-1	*Eryngium foetidum*	PP840582.1	PP841284.1	PP841289.1	PP841304.1
C. syngoniicola	LC8896	*Syngonium sp*	MZ595865.1	MZ664163.1	MZ799298.1	MZ664119.1
C. vittalense	QZ-6	*Theobroma cacao*	PP690784.1	PQ007591.1	PQ259888.1	PP977497.1
Colletotrichum gigasporum species complex						

C. arxii	CBS:132511	*Paphiopedilum x lanthanium*	KF687716.1	KF687802.1	KF687780.1	KF687843.1
C. durionigenum	MFLUCC 22-0111	fresh durian fruit	OP740244.1	OP744503.1	OP744504.1	OP744505.1
C. gigasporum	CBS:133266	*Centella asiatica*	KF687715.1	PP444995.1	KF687761.1	KF687822.1
	**BEC191A**	***Coffea arabica***	**PP838109**	**C_AA076831.1**	**C_AA076979.1**	**C_AA077053.1**
C. jishouense	GZU_HJ2 G3	*Nothapodytes pittosporoides*	MH482929.1	MH708137.1	----	MH492706.1
C. magnisporum	CBS:398.84	*Stemona japonica (Bl.)* Miq	KF687718.1	KF687803.1	KF687782.1	KF687842.1
C. pseudomajus	CBS:571.88	*Camellia sinensis*	JX009424.1	KF687801.1	KF687779.1	KF687826.1
C. radicis	CBS:529.93	unidentified plant	KF687719.1	KF687785.1	KF687762.1	KF687825.1
C. subvariabile	LC13876	unidentified plant	MZ595883.1	MZ664181.1	MZ799343.1	MZ664054.1
C. variabile	LC13875	unidentified plant	MZ595884.1	MZ664182.1	MZ799344.1	MZ664055.1
C. vietnamense	CBS:125478	unidentified plant	MH863700.1	KF687792.1	KF687769.1	KF687832.1
C. zhaoqingense	LC13878	unidentified plant	MZ595906.1	MZ664204.1	MZ799305.1	MZ664066.1
Colletotrichum gloeosporioides	CBS 119204	*Pueraria montana var.* lobata	JX010150.1	JX009502.1	JX009790.1	JX010013.1
Colletotrichum acutatum	CBS:112996	*Carica papaya*	JQ005776.1	JQ005839.1	JQ005797.1	JQ948677.1
Colletotrichum destructivum	CBS:136228	*Trifolium hybridum*	KM105207.1	KM105417.1	KM105277.1	KM105561.1
